# Retraction: Inhibition of protein phosphatase 2A with a small molecule LB100 radiosensitizes nasopharyngeal carcinoma xenografts by inducing mitotic catastrophe and blocking DNA damage repair

**DOI:** 10.18632/oncotarget.28727

**Published:** 2025-05-19

**Authors:** Peng Lv, Yue Wang, Jie Ma, Zheng Wang, Jing-Li Li, Christopher S. Hong, Zhengping Zhuang, Yi-Xin Zeng

**Affiliations:** ^1^Cancer Institute and Hospital, Chinese Academy of Medical Sciences (CAMS), Beijing, People’s Republic of China; ^2^Beijing Neurosurgical Institute, Capital Medical University, Beijing, People’s Republic of China; ^3^Institute for Medical Device Standardization Administration, National Institutes for Food and Drug Control, Beijing, People’s Republic of China; ^4^Surgical Neurology Branch, National Institute of Neurological Disorders and Stroke, National Institutes of Health, Bethesda, MD, USA; ^5^Department of Experimental Research, Sun Yat-sen University Cancer Center, Guangzhou, Guangdong, People’s Republic of China; ^5^State Key Laboratory of Oncology in Southern China, Guangzhou, Guangdong, People’s Republic of China

**This article has been retracted:** Oncotarget has completed its investigation of this paper. Our review identified several instances of duplicated western blot images within Figure 7. The first author, Peng Lv, has acknowledged these errors and has requested a retraction of the paper on behalf of all co-authors. In light of these facts, the Editorial decision was made to retract this paper. All authors agreed with the decision.

Original article: Oncotarget. 2014; 5:7512–7524. 7512-7524. https://doi.org/10.18632/oncotarget.2258

